# Surface Electromyography Signal Processing and Classification Techniques

**DOI:** 10.3390/s130912431

**Published:** 2013-09-17

**Authors:** Rubana H. Chowdhury, Mamun B. I. Reaz, Mohd Alauddin Bin Mohd Ali, Ashrif A. A. Bakar, Kalaivani Chellappan, Tae. G. Chang

**Affiliations:** 1 Department of Electrical, Electronic and Systems Engineering, Universiti Kebangsaan Malaysia, Bangi, Selangor 43600, Malaysia; E-Mails: mamun.reaz@gmail.com (M.B.I.R.); mama@eng.ukm.my (M.A.B.M.A.); ashrif@eng.ukm.my (A.A.A.B.); kckalai@ukm.my (K.C.); 2 School of Electrical and Electronics Engineering, Chung Ang University, 221 Hueksuk-Dong, Dongjak-Gu, Seoul 156-756; Korea, E-Mail: tgchang@cau.ac.kr

**Keywords:** electromyography, noise source, wavelet, EMD, ICA, artificial neural network, HOS

## Abstract

Electromyography (EMG) signals are becoming increasingly important in many applications, including clinical/biomedical, prosthesis or rehabilitation devices, human machine interactions, and more. However, noisy EMG signals are the major hurdles to be overcome in order to achieve improved performance in the above applications. Detection, processing and classification analysis in electromyography (EMG) is very desirable because it allows a more standardized and precise evaluation of the neurophysiological, rehabitational and assistive technological findings. This paper reviews two prominent areas; first: the pre-processing method for eliminating possible artifacts via appropriate preparation at the time of recording EMG signals, and second: a brief explanation of the different methods for processing and classifying EMG signals. This study then compares the numerous methods of analyzing EMG signals, in terms of their performance. The crux of this paper is to review the most recent developments and research studies related to the issues mentioned above.

## 1. Introduction

Electromyograpy (EMG) refers to the collective electric signal from muscles, which is controlled by the nervous system and produced during muscle contraction. The signal represents the anatomical and physiological properties of muscles; in fact, an EMG signal is the electrical activity of a muscle's motor units, which consist of two types: surface EMG, and intramuscular EMG [1]. Surface EMG and intramuscular EMG signals are recorded by non-invasive electrodes and invasive electrodes, respectively. These days, surface-detected signals are preferably used to obtain information about the time or intensity of superficial muscle activation [2]. Electromyography (EMG) signals are considered most useful as electrophysiological signals in both medical and engineering fields. The basic method for understanding the human body's behaviors under normal and pathological conditions is provided by the recording of EMG signals. Whenever an EMG signal is being recorded from the muscle, various types of noises contaminate it. Therefore, analyzing and classifying the EMG signals is very difficult because of the complicated pattern of the EMG, especially when EMG motion occurs [3]. EMG signals can be used to generate device control commands for rehabilitation equipment such as robotic prostheses and in generic man-machine interfaces for Human Computer Interface (HCI). They have also been deployed in many clinical and industrial applications [4]. Processing and classifying EMG signals requires using the Electromyographic Control technique. Control systems based on the classification of EMG signals are usually known as Myoelectric Control Systems (MCSs); powered upper-limb prostheses and electric-powered wheelchairs are two of the main potential applications of MCSs [5]. However, to use these applications effectively, an accurate EMG signal acquisition is a pre-requisite. When acquiring an EMG signal, various background noises are received due to the presence of electronic equipment and physiological factors. Section 1 of this paper provides an overview of these various noises and mentions ways to overcome them (when the acquisition of an EMG signal is completed). Nevertheless, it remains very difficult for the noise to be removed clearly. Therefore, that EMG signal is processed and analyzed to get the required information. Many researchers have used different kinds of advanced methodologies, including wavelet transform, Wigner-Ville Distribution, Independent component analysis, Empirical mode decomposition, and higher-order statistics for analyzing the EMG signal appropriately. The second section of this paper contains EMG signal classification methods.

## 2. Noise Sources in EMG Signals

The identity of an actual EMG signal that originates in the muscle is lost due to the mixing of various noise signals or artifacts. The attributes of the EMG signal depend on the internal structure of the subject, including the individual skin formation, blood flow velocity, measured skin temperatures, the tissue structure (muscle, fat, *etc.*), the measuring site, and more. These attributes produce different types of noise signals that can be found within the EMG signals. This may have an effect on the result of feature extraction and hence affect the diagnosis of the EMG signals. Various methods of noise elimination have been proposed during the EMG signal acquisition, and the subject continues to be a popular one among practitioners. The main challenges in analyzing the EMG signals are explained below.

### 2.1. Inherent Noise in the Electrode

All types of electronic equipment generate electrical noise, otherwise known as “inherent noise”. This noise has frequency components that range from 0 Hz to several thousand Hz. Two kinds of EMG signals in widespread use include surface EMG, and intramuscular (needle and fine-wire) EMG. To perform intramuscular EMG, a needle electrode or a needle containing two fine-wire electrodes is placed within the muscle of interest (invasive electrode). However, the use of surface electrodes has become more accepted in clinical and physiological applications [6]. The advantage of surface electrodes is that they are non-invasive, and the patient need not be anesthetized before placing the electrode. The operation is simple and painless.

For recording the EMG, the non-invasive electrodes are applied to the skin of the subject. For recording purposes, electrodes made of silver/silver chloride (10 × 1 mm) have been found to give adequate signal-to-noise ratio and are electrically very steady. For this reason, they are widely used as surface electrodes [7]. When the electrode size enlarges, the impedance decreases. However, electrode size should not be very large. On the other hand, high electrode impendence effectively reduces the signal quality and gives low signal-to-noise ratio. Therefore, both parameters should be taken into consideration. Researchers are allowed to use high electrode impedances for experiments in which statistical power is high or in which large numbers of electrodes are necessary, but tend to switch to low electrode impedances for experiments in which statistical power would otherwise be too low [8]. This noise can be eliminated by using intelligent circuit design and high quality instruments.

### 2.2. Movement Artifact

Movement of the cable connecting the electrode to the amplifier and the interface between the detection surface of the electrode and the skin creates motion artifacts. Muscle fibers generate electric activity whenever muscles are active [9]. EMG signals are recorded by placing electrodes close to the muscle groups. When the muscle is activated, the length of the muscle decreases and the muscle, skin and electrodes move with respect to one another. At that time, the electrodes will show some movement artifacts. The frequency range of the motion noise is usually 1–10 Hz and has a voltage comparable to the amplitude of the EMG. Recessed electrodes can remove the movement artifact significantly, in which a conductive gel layer is used between the skin surface and the electrode-electrolyte interface. Another type of movement artifact occurs due to the potential difference between skin layers. Recessed electrodes cannot remove this artifact. However, this type of artifact is attenuated by reducing the skin impedance [10]. Tam and Webster [11] found that scratching the skin reduces these artifacts. Burbank and Webster [12] showed that low skin impedance could be achieved by using the puncture technique, thus reducing the artifacts. Conforto *et al.* [13] tested four filtering procedures to reject the motion artifact from an EMG signal during dynamic contractions. These procedures include the eighth order Chebyshev high pass filters with corner frequency at 20 Hz; the moving average filter; the moving median filter; and the adaptive filter, which is based on orthogonal Meyer wavelets. They found that the wavelet procedure maintains all the information and detects the time more precisely than the other methods. The virtual movement between skin surface electrodes and the innervations zone(s) of the underlying motor units can cause another type of motion artifact. Mesin *et al.* discovered that the outcome of the innervations zone (IZ) on amplitude, frequency and conduction velocity can be calculated from the EMG and the effect of electrodes placed close to IZ. At the same time, they showed that the inter-electrode distance must be thin with respect to the distance between the IZ and the tendon, and that no electrode should go beyond this zone [14].

### 2.3. Electromagnetic Noise

The human body behaves like an antenna—the surface of the body is continuously inundated with electric and magnetic radiation, which is the source of electromagnetic noise. Electromagnetic sources from the environment superimpose the unwanted signal, or cancel the signal being recorded from a muscle. The amplitude of the ambient noise (electromagnetic radiation) is sometimes one to three times greater than the EMG signal of interest.

The human body's surface continuously emits electromagnetic radiation, and avoiding exposure to ambient noise on the surface of the Earth is impracticable [15]. The dominant concern for the ambient noise arises from the 60 Hz (or 50 Hz) radiation from power sources, which is also called Power-Line Interference (PLI). This is caused by differences in the electrode impedances and in stray currents through the patient and the cables. However, in order to remove the recorded artifact, off-line processing is necessary [10]. A high pass filter can remove the interference if the frequency of this interference is high. However, if the frequency content of PLI is within the EMG signal then it is very essential to recognize the nature of the EMG signal. 50 Hz PLI and its four harmonics (e.g., 100, 200, 300 and 400 Hz) are constructed mathematically by the equation [16]:

(1) $${\text{PLI}}_{\text{ref}}=\text{cos}(2\pi \;50t)+\text{cos}(2\pi \;100t)+\text{cos}(2\pi \;200t)+\text{cos}(2\pi \;300t)+\text{cos}(2\pi \;400t)$$

**Figure 1.**
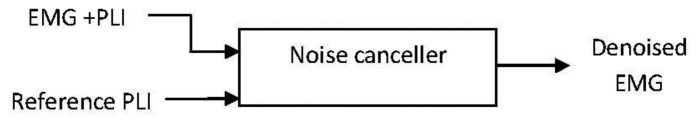
General block diagram of PLI cancelling system.

Figure 1 illustrates the general model for the PLI cancellation system. A number of adaptive filter techniques have been proposed for the attenuation of the PLI noise, such as adaptive FIR notch filter, adaptive IIR notch filter, adaptive notch filter using Fourier transform and so forth. An efficient Laguerre filter can eliminate power-line interference from EMG signals successfully; in fact, it has been shown to be more effective than other adaptive algorithms. This filter can increase the SNR of an EMG signal significantly without using any information from the power-line interference [16].

### 2.4. Cross Talk

An undesired EMG signal from a muscle group that is not commonly monitored is called “crosstalk”. Crosstalk contaminates the signal and can cause an incorrect interpretation of the signal information [17]. Crosstalk depends on the many physiological parameters [18,19], and can be minimized by choosing electrode size and inter-electrode distances (typically 1–2 cm or the radius of the electrode) carefully. Electrodes with a smaller surface area reduce bipolar spacing and mathematical differentiation, and the combination of these three methods reduces the potential crosstalk effectively [20]. Crosstalk increases with increasing subcutaneous fat thickness. Lowery *et al.* showed that the distance from the active fibers increases the decay rate of the cross-correlation function, and acts faster than crosstalk. The cross-correlation between two EMG signals is neither a qualitative nor a quantitative measure of crosstalk [19]. The main causal factor of crosstalk is the generation of the non-propagating signal components due to loss of the intracellular action potentials at the tendons. Thus, crosstalk has a different shape with respect to the signals detected directly over an active muscle and has a broader bandwidth than these signals. The cross-correlation coefficient analysis and high pass filtering method have no effect on crosstalk and are not reliable for reducing it [21]. Selectivity of EMG electrodes depends on their interspacing, their conductive area, and axis direction with respect to the direction of the underlying muscular fibers. Minimal crosstalk area (MCA) is defined as a surface where crosstalk versus co-contraction of muscles is minimal. The precise location and measurements of the distance between two bony landmarks are the keys to finding the “minimal crosstalk area” of the targeted muscle. MCA helps to limit or avoid crosstalk from neighboring muscles [22].

Mezzarane *et al.* presented the mathematical relationship (see Equation (2) below) between the target muscle EMG and crosstalk [23]:

(2) $${\text{T}}_{\text{b}}^{2}={\text{Rb}}_{\text{c}}^{2}+{\text{T}}_{\text{i}}^{2}+{\text{Ob}}_{\text{c}}^{2}$$

where, the background EMG activity recorded at the target muscle = Tb, the intrinsic activity of the target muscle itself = Ti, the crosstalk from the remote muscle = Rb and the crosstalk from other muscles = Ob. These random signals, Ti, Rb and Ob are assumed to be uncorrelated; hence, the variance of Tb is the sum of the variances of Ti, Rb and Ob.

### 2.5. Internal Noise

Anatomical, biochemical and physiological factors take place due to the number of muscle fibers per unit, depth and location of active fibers, and amount of tissue. These factors are called internal noise and directly affect EMG signal quality. Conventionally, physical capacitive effects are assumed negligible when analyzing the EMG signals. However, these assumptions might not be valid f or muscle tissue. Both muscle conductivity and permittivity are frequency-dependent (dispersive). Furthermore, skin has a relatively low conductivity and high permittivity such that capacitive effects would be expected to be significant and the dispersive effects of permittivity will be more pronounced [24]. Therefore, the capacitive effects also act as an internal noise for an EMG signal. The amount of the tissue between contracting muscles and electrodes, along with their thickness, affect the amplitude of the EMG signal. Hemingway *et al.* showed that if the thickness of the subcutaneous tissue between the surface electrode and active muscles increases, then the electromyographic activity decreases [25]. They observed the effect by examining 20 normal subjects who contracted their muscle force for 45 s. It should be mentioned that all the subjects had varying amounts of subcutaneous tissue. The amount of excess body fat is considered as an internal noise for EMG because it increases the separation between the active muscle fibers and the detection sites. Under the recording sites, surgical fat layer reduction increases surface EMG signal amplitude [26]. These effects can be partly reduced by using high pass spatial filters [27].

### 2.6. Inherent Instability of the Signal

The amplitude of the EMG signal is quasi-random in nature. The frequency components between 0 and 20 Hz are mostly unstable because they are affected by the firing rate of the motor units. The firing rate of the motor units is quasi-random in nature. Because of the unstable nature of these components of the signal, it is considered as unwanted noise. The numbers of active motor units, motor firing rate and mechanical interaction between muscle fibers can change the behavior of the information in the EMG signal [15].

### 2.7. Electrocardiographic (ECG) Artifacts

The electrical activity of the heart is the foremost interfering component for surface electromyography (sEMG) in the shoulder girdle, which is called an “electrocardiogram (ECG) artifact” [28]. Cardiac activity (ECG artifact) often contaminates EMG signals, especially in trunk muscle electromyography [29]. The placement of EMG electrodes, which is conducted by a selection of the pathological muscle group, often decides the level of ECG contamination in EMGs. ECG contamination in EMGs may be kept at a minimal level by common-mode rejection at the recording site, by the careful placement of bipolar recording electrodes along the heart's axis if possible [30]. Due to an overlap of frequency spectra by ECG and EMG signals and their relative characteristics, such as non-stationarity and varied temporal shape, it is very difficult to remove the ECG artifacts from the EMG signal [31]. ECG contamination is only visually identifiable below 25% maximum voluntary contraction (MVC) of EMG activation. However, Hu *et al.* suggested that the level of corruption by ECG artifacts on sEMG parameters is more serious and prominent under static sEMG measurements [32]. High-pass filtering at 100 Hz essentially removed the effect of ECG interference. Whenever subjects are maintaining constant force contraction, repetitive fluctuation occurs in the intensity of surface EMG signals due to the ECG artifact. High-pass filter is a very effective method to eliminate this oscillation caused by the ECG artifact [33].

## 3. EMG Signal Processing

In the field of clinical diagnosis and biomedics, the analysis of EMG signals with powerful and advanced methodologies is becoming more and more a required tool for healthcare providers. This overview covers recent advances in the field of EMG signal processing.

### 3.1. Wavelet Analysis

The time-frequency plane is one of the most fundamental concepts in signal analysis. The Wigner-ville distribution (WVD) is one time-frequency representation method, which is used for analyzing the EMG signal. In 1992, Ricamato *et al.* showed that it is possible to present the frequency ranges of the motor unit by WVD [34]. WVD is highly concentrated in the instantaneous frequency and time of the signal, which is an excellent localization property of this method. It has a cross-term effect and is very noisy. Therefore, it is not well suited for analyzing a multi-component signal like EMG.

Wavelets have been growing in popularity as an alternative to the usual Fourier transform method. The wavelet transform can essentially be divided into discrete and continuous forms. It efficiently transforms the signals with a flexible resolution in both time- and frequency-domains. The time taken for processing the signal using Discrete Wavelet Transform (DWT) method is low. However, in Continuous Wavelet Transform (CWT), it is more consistent and less time-consuming due to the absence of down sampling. The DWT method has been successful in analyzing non-stationary signals, such as surface EMG (sEMG) signals, but it yields a high-dimensional feature vector [35].

The basic analytical expression for CWT is presented in Equation (3) below. In a wavelet transform, the wavelet corresponding to scale *a* and time location *b* is given by:

(3) $$\psi \left(a,b\right)=\overset{1}{\;}/\underset{\sqrt{\left|a\right|}}{\;}\psi (\frac{t-b}{a})$$

where *ψ*(t) is the ‘mother wavelet’ which can be taken as a band-pass function. The factor $\sqrt{\left|\text{a}\right|}$ is used to ensure energy preservation, which is the same for all values of a and b. There are various ways of discretizing timescale parameters (a, b), and each one yields a different type of wavelet transform.

Successive low-pass and high pass filtering in the discrete-time domain computes the DWT. The general equation of DWT (Equation (4)), is given below:

(4) $$x\left(t\right)=\sum_{k=-\infty }^{\infty }\sum_{l=-\infty }^{\infty }d\left(k,l\right)2^{\frac{k}{2}}\psi (2^{-k}t-1)$$

where *k* is related to *a* as: *a* = *2k*; *b* is related to *l* as *b* = *2kl*; and *d (k,l)* is a sampling of *W* (*a*,*b*) at discrete points *k* and *l*.

Daubechies analyzed the time series that contained non-stationary power at many different frequencies, by using wavelet transform [36]. The different types of wavelets have different time-frequency structures. There are several factors that should be considered when choosing the wavelet function [37]. Guglielminotti and Merletti theorized that wavelet transform exhibits very good energy localization in the time-scale plane when the shape of the MUAP is matched with wavelet shape [38], in the order that the wavelets are generally chosen, whose shapes are similar to those of the MUAP. In 1997, Laterza and Olmo explained that wavelet transform was developed as an alternative approach to other time frequency representations to overcome the resolution problem. Moreover, WT is not affected by cross terms, which is particularly relevant when dealing with multi-component signals [39]. The WT is principally useful for MUAP detection in the presence of additive white noise. Mexican hat wavelet and the Morlet wavelet are the most popular continuous wavelets. One of the disadvantages in this approach is that the Mexican hat wavelet does not accurately match the MUAP shape. The major problem of fast and short term Fourier transform (SFT and FFT) is that the signals are considered to be stationary signals [40]. Therefore, to overcome this problem Pattichis and Pattichis processed the signal at different resolution levels by using the continuous wavelet transform [41].

The pre-processing stage based on a wavelet de-noising algorithm for sEMG upper- and lower-limb movement recognitions has been a huge success over the past few years [42,43]. Removing the interference of random noises from EMG signals (for example, white Gaussian noise (WGN)) using filtering procedures is difficult. Wavelet de-noising algorithms can effectively remove these noises [44]. Phinyomark *et al.* provided the basic idea of a wavelet-based de-noising procedure. The application of this algorithm needs the selection of five processing parameters, including: (1) the type of wavelet basis function; (2) the scale; (3) the threshold selection rule; (4) the threshold rescaling method, and (5) the thresholding function [44]. Selecting the right wavelet function is the most crucial part of wavelet de-noising, which in turn depends on a number of factors, such as the type of application and characteristics of the signal. Phinyomark *et al.* studied five wavelet functions (db2, db5, sym5, sym8 and coif5) for de-noising the sEMG signal for multifunction myoelectric control. They analyzed the processed sEMG by measuring the mean square error (MSE) parameter and showed that the scale level 4 provides the better performance when compared with other scale levels. They also showed that the fifth order of Coiflet provides the perfect reconstruction for sEMG signal [45]. Where the signals contain discontinuities and sharp spikes, the wavelet transform de-noising method finely preserved the maximum signal character [46]. The selection of suitable wavelet functions from three twenty-four wavelet function and decomposition levels is very important for EMG signal from a de-noising viewpoint. Jiang and Kuo assessed four classical threshold estimation functions and concluded that EMG signals are insensitive to the selection of threshold estimation functions [47]. In 2003, Kumar *et al.* determined muscle failure by using the Symlet function (Sym4 and Sym5) with decomposition level 8 and 9 (out of 10 levels) [48]. Hossain and Mamun proved that WF db45 shows the best contrast when they analyzed the sEMG signal using both power spectrum and bispectrum compared to the other four WFs (Haar, db2 sym4 and sym5) within the range 50 to 70 Hz [49]. In 2012 Wei *et al.* proposed a new wavelet-based algorithm that analyzed surface EMG signals in three steps [50]. For de-noising EMG, they applied a Maximal Overlap Discrete Wavelet Transform (MODWT) algorithm and decomposed EMG data into different frequency band oscillations. For this algorithm they used the wavelet function db4 at decomposition level 5. It was an easy, simple and inexpensive process.

The benefit of using a wavelet basic function is that it has continuous derivatives, which allows it to decompose a continuous function more efficiently. It also avoids unwanted signals. Daubechies's wavelets provide better energy concentration with long-length filters than those with short-length filters [51]. Table 1 displays the different types of wavelet functions with their families.

**Figure 2.**
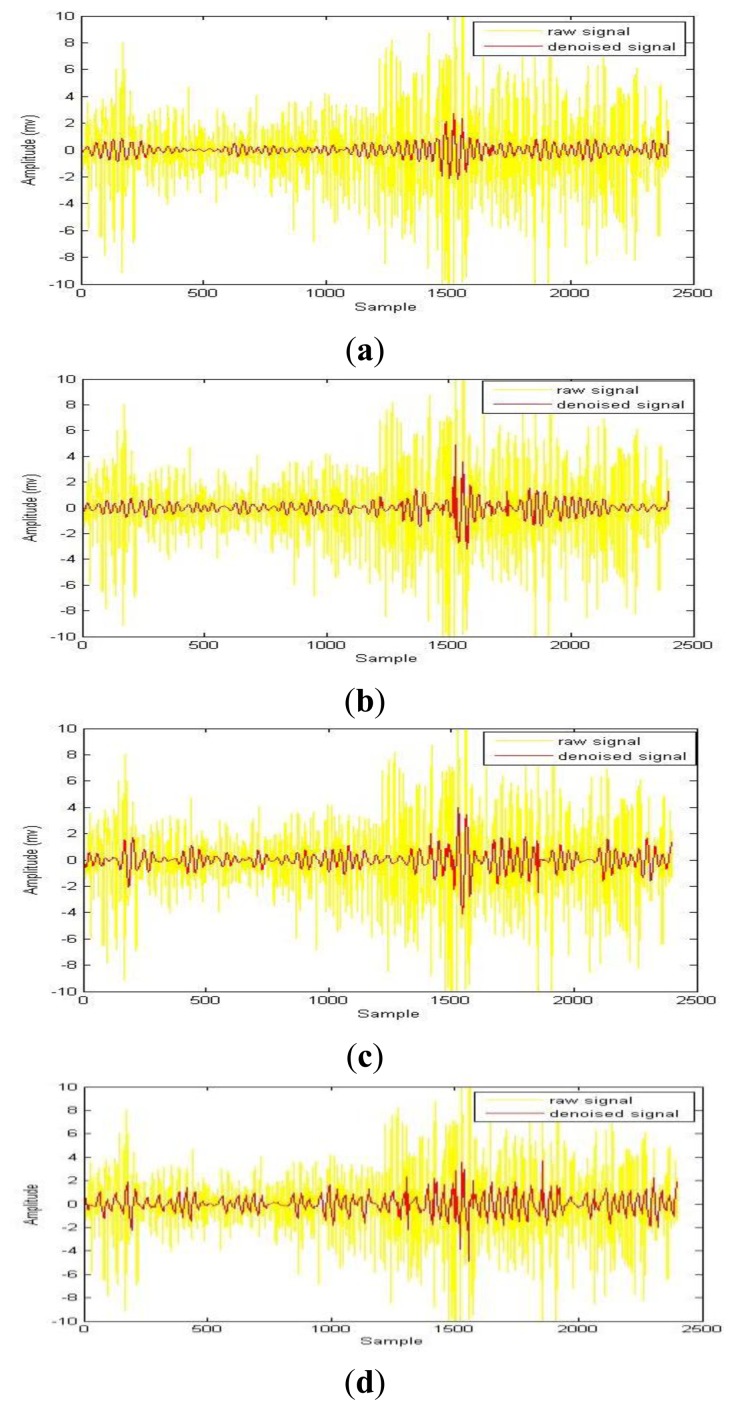

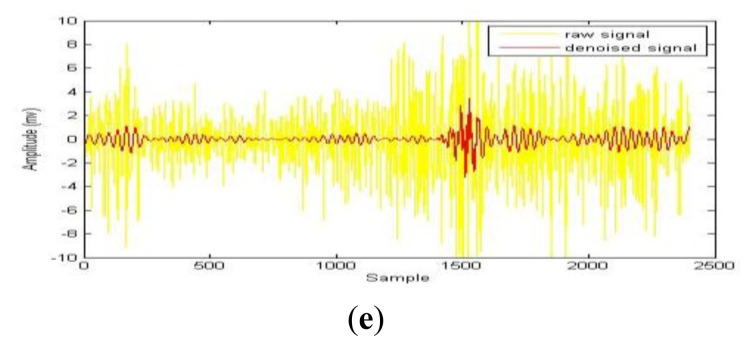
Raw EMG signal denoised by wavelet function (**a**) db2; (**b**) db4; (**c**) db6; (**d**) db44; (**e**) db45.

By investigating and analyzing various research studies on wavelet transform, the author has concluded that analyzing sEMG signals using Daubechies's function renders successful results. For obtaining better results from a sEMG analysis on different applications, the author recommends to use the db function (db2, db4, db6, db44 and db45) at decomposition level 4. In case of high and low noises in sEMG, the db function at decomposition level 4 can be used as a compromise level. The author simulated the raw sEMG signal by using the above wavelet functions. Figure 2 represents the raw sEMG signal from the right *rectus femoris* muscle during maximum walking speed and its de-noised version using a different wavelet function, such as db2, db4, db6, db44 and db45 at decomposition level 4.

### 3.2. Higher Order Statistics (HOS)

Higher order spectra are defined as spectral representations of higher order cumulants of a random process. Let x (k) be a real, discrete time and nth-order stationary random process. Moreover, let w = [w1, w2…wn] T and *x* = [*x*(*k* + *τ_1_*),……,*x*(*k* + *τ_n_*_−1_)]*^T^*. Then the nth-order moment of *x* (*k*), ${\text{M}}_{\text{n}}^{\text{x}}=\left(\tau_{1},\tau_{2}\cdots \tau_{n-1}\right)$ is defined as the coefficient in the Taylor expansion of the moment generating function in Equation (5):

(5) Φ(W)=E[exp(iWTx)]

In practice, the nth-order moment can be equivalently calculated by taking an probability over the process multiplied by (n−1) lagged versions of itself. Higher order spectra are often estimated directly in the spectral domain as expected values of higher order periodograms. The spectral representation of Higher Order Statistics (HOS), such as moments and cumulants of the third order and above, are known as polyspectra or higher order spectra. For efficient processing of the EMG signal, HOS is applicable due to its unique properties. HOS can identify deviations from linearity, stationarity or Gaussianity in the signal [52]. HOS is important for a quality neuromuscular diagnosis to obtain information on innervation pulse trains and Motor-Unit Action Potentials (MUAPs) characteristics. Kanosue *et al.* developed a statistical signal processing method that can determine the amplitude and number of recruited MUAPs [53]. The spectral moments (second and fourth order) with a parametric model are used in this method. Second-order statistics (SOS) provide low order models and present the real data that is parsimonious with the particle dimension. Within the past few decades, there has been considerable interest in using higher order statistics (HOS) technique [15]. HOS was introduced in the 1960s, and Giannakis and Tsatsanis applied HOS for EMG signal analysis in 1991. The advantage of HOS is that accurate phase reconstruction is possible in the HOS domain, but SOS is phase-blind [54]. Moreover, HOS is useful for modelling non-Gaussian and nonlinear processes. Kalpanis *et al.* gave the theory using HOS, which characterizes the Gaussianity of the sEMG signal by using the bicoherence index. sEMG signal distribution is highly non-Gaussian at low and high levels of force, whereas the distribution has its maximum Gaussianity at the mid-level of maximum voluntary contraction (MVC). They used the HOS technique in their sEMG signal analysis in order to extract a new parameter (power spectrum median frequency) that could enhance the diagnostic character of sEMG [55]. In probability theory and statistics, the skewness (measure of third order cumulants) measures asymmetry and kurtosis (measure of fourth order cumulants) measures peakness of the probability distribution. Cumulants and moments are particularly convenient; this is why cumulants and moments are successfully used in the higher order statistics technique. In an earlier stage, Yana *et al.* used HOS-based approaches to recover MUAPs from the sEMG signal [56]. However, this approach was only applied to simulated sEMG signals. Shahid *et al.* applied HOS to the EMG signal and proposed the ‘Bispectrum of Linear Systems’ to characterize the motor unit action potential due to its advantages of HOS over SOS [57]. The EMG processing method based on the first and second order moments and cumulants (SOS) cannot suppress white Gaussian noise from the signal where HOS (bispectrum or third order spectrum) can eliminate this noise. The mathematical model of the EMG signal is of the output of a Linear Time Invariant (LTI) system whose input is non-Gaussian white noise. Using the convolution theorem for the LTI system, the output x (n) can be expressed as:

(6) $$x\left(n\right)=\sum_{k=0}^{\alpha }h\left(k\right)e\left(n-k\right)+w\left(n\right)$$

where w (n) is an independent identically distributed random-Gaussian white noise; e (k) is a white non-Gaussian process; and h (k) is a stable, possibly nonlinear kernel representing the EMG segment x (n). Based on this model, they applied cepstrum of bispectrum-based system reconstruction algorithm on the real EMG for estimating the appearance of MUAPs when the muscles were at different contraction positions. Bispectrum is a part of the family of higher-order spectra. Due to the speedy and economical software-based solution to visualizing MUAPs, this algorithm can regain high-quality estimates of MUAPs from sEMG signals. However, this technique does not have a capacity to detect the effect of increased loading and exertion by the muscle.

Whenever a signal-processing technique is applied on the diagnosis of neuromuscular disorders, some parameters, such as amplitude, number of phases, spike duration, number of turns, *etc.* should be taken into consideration. The HOS method characterizes and detects the non-linearity of the sEMG signal. This method is also able to estimate both the amplitude and phase information successfully. From the above analysis of various research works on the HOS technique, the author concluded that this method is more useful for analyzing the sEMG signal in case of diagnosing neuromuscular disorder.

### 3.3. Empirical Mode Decomposition (EMD)

EMD is a moderately new, data-driven adaptive technique for the analysis of non-stationary and nonlinear signals. EMD is a method to analyze the underlying notion of instantaneous frequency, and provides insight into the time-frequency signal features. The EMD method was first introduced by Huang *et al.* [58], and is used as a sifting process that estimates intrinsic mode functions (IMFs).

EMD aims to decompose a multi-component signal, x(t) into a number of virtually mono-component IMFs, h(t) plus a non-zero-mean value of the residual component, r(t):

(7) $$\text{x}\left(\text{t}\right)=\sum_{\text{i}=1}^{\text{h}}{\text{h}}^{\left(\text{i}\right)}\left(\text{t}\right)+\text{r}(\text{t})$$

Each one of the IMFs; e.g., h(k + 1), is obtained by applying a process called sifting to the residual multi-component signal as in the following Equation (8):

(8) $${\text{x}}^{\left(\text{k}\right)}\left(\text{t}\right)=\text{x}\left(\text{t}\right)-\sum_{\text{i}=1}^{\text{k}}{\text{h}}^{\text{i}}(\text{t})$$

The sifting process is an iterative procedure which aims to achieve improved estimates of hk(t) in each iteration. More specifically, during the (n + 1) th sifting iteration, the temporal estimate of the IMF hnk(t), is obtained in the previous sifting iteration. This process is repeated until the designated IMF fulfills the following criteria:

(1) The number of extrema and the number of zero crossings must either equal one another, or differ at most by one.
(2) The mean value of the upper envelope and lower envelope is zero at any point of the whole time series.

**Figure 3.**
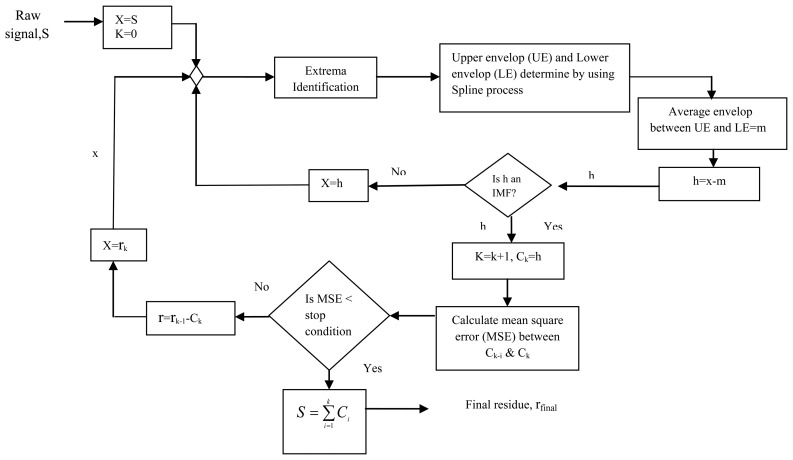
Block diagram of Empirical Mode Decomposition [55].

When the IMF component is a monotonic function, the process is finalized and the original signal is reconstructed by adding all the IMF components along with the mean of final residue, m_final_. Final residue is obtained by the difference between S(t) and the sum of all IMFs. The reconstructed signal, S(t) can be represented as in the following Equation (9):

(9) $$S\left(t\right)=\sum_{k=1}^{n}{\text{IMF}}_{n}+m_{\text{final}}$$

where n is the number of IMFs. Adriano *et al.* first used the EMD signal processing technique for filtering electromyographic (EMG) signals that can decompose an EMG signal into a set of IMFs [59]. The sequence of steps for estimating the intrinsic mode functions of the EMD process is given in Figure 3.

**Figure 4.**
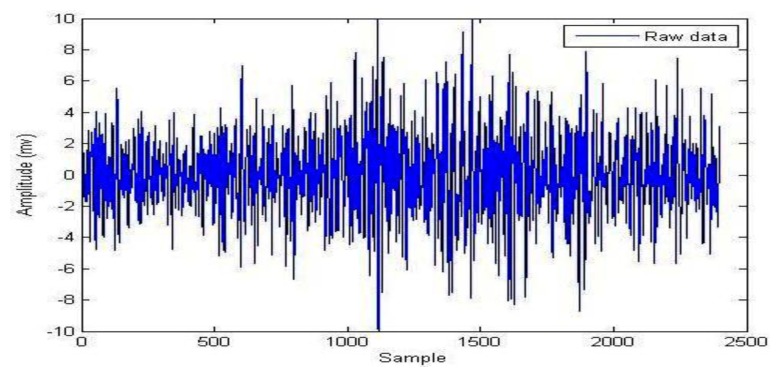
Raw EMG data from right *vastus medialis* muscle during maximum walking speed.

**Figure 5.**
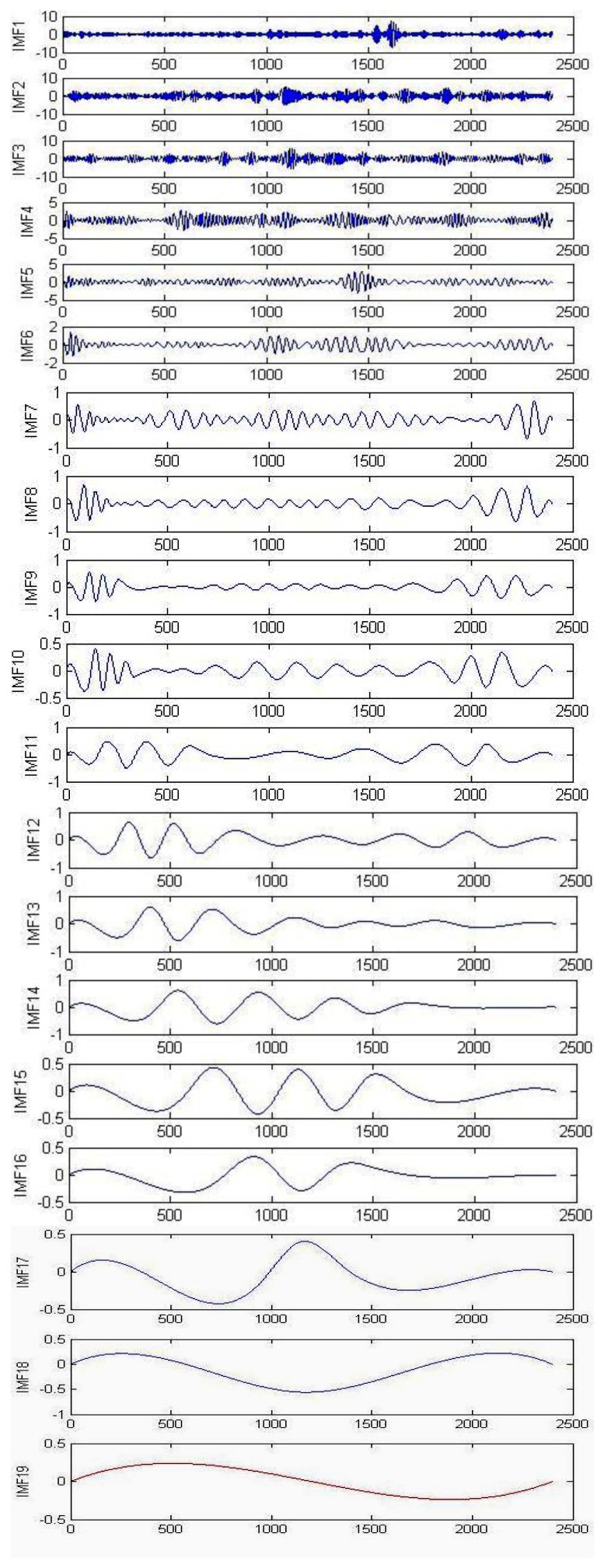
The empirical mode decomposition of the electromyography signal from right *vastus medialis* during maximum walking speed.

During the signal processing, EMG signals use the EMD for background activity attenuation. EMD is very effective for noise reduction because it is a non-linear method that can deal with non-stationary data. This procedure makes no assumptions about the input time-series where the wavelet procedure depends on the basic mother wavelet function. Andrade *et al.* showed that the EMD method provides better results for the attenuation of EMG background activity (noise) when compared with different wavelet prototypes (db2, db3 and db4). However, computing IMFs takes a lot of time, which can be disadvantageous when compared to wavelets [59]. Figure 4 illustrates the raw EMG data from the right vastus medialis muscle during maximum walking speeds; EMD decomposes it into a finite number of intrinsic mode functions, which is shown in Figure 5.

The major drawback of the EMD method is that it is more sensitive to the presence of noise, and has a mode-mixing problem. The EMD method is also a time-consuming process. Therefore, a more robust, noise-assisted version of the EMD algorithm, called Ensemble EMD (EEMD) is used [60]. Ensemble EMD (EEMD) was introduced to remove the mode-mixing effect. The EEMD bypasses the mode-mixing problem of the original EMD by adding white noise into the targeted signal repeatedly. It also provides physically unique decompositions when it is applied to data with mixed and intermittent scales. Zhang *et al.* showed that different types of noises [power-line interference (PLI), white Gaussian noise (WGN), and baseline wandering (BW)] could be adaptively removed based on an IMF filtering where an EMD/EEMD-based IMF filtering framework achieved improved performance than the conventional digital filters (IIR causal and IIR non-causal filters). It has been found that with a low level of SNR of the processed signal, the EEMD method provided the best surface EMG de-noising performance compared to all other methods [61].

By studying the sEMG signal analysis using the empirical mode decomposition technique, the author has come to the conclusion that the EEMD method offers the most successful results for the attenuation of specific noises of sEMG signals. This method is more robust and the filtering procedure is able to directly extract signal components, which overlap significantly in time and frequency. EEMD achieved best surface EMG de-noising performance for attenuating noises, especially in cases of power-line noises (PLI), white Gaussian noise (WGN), baseline wandering (BW) and ECG artifacts.

### 3.4. Artificial Neural Network (ANN)

The Neural Network (NN) approach is suitable for modeling nonlinear data and is able to cover distinctions among different conditions. The requirements for designing an ANN for a given application include: (i) determining the network architecture; (ii) defining the total number of layers, the number of hidden units in the middle layers and number of units in the input and output layers; and (iii) the training algorithm used during the learning phase [62]. The back propagation neural network (BPNN) is a popular learning algorithm to train self-learning feed-forward neural networks. However, some drawbacks of this method exist, in that NN requires a huge quantity of training data, the network architecture is quite rigid and NN takes too many learning iterations [63]. Another neural network, called Cascade Correlation Network (CCN), can overcome the limitations of BPNN. It reduces the Mean Square value of the required signal and the convergence time, while increasing the SNR. CCN is an architecture that uses a supervised learning algorithm for artificial neural networks [64]. CCN offers several advantages, such as: no need to guess the size, depth and connectivity pattern of the network in advance; it learns approximately 50 times faster than a standard back-propagation algorithm; and it is very appropriate for large training sets. However, a maximum correlation criterion systematically pushes the hidden layers to their saturated excessive values in place of an active layer, so the error surface becomes rough. This is the main disadvantage of CCN [65]. Mankar and Ghatol mentioned that a radial basis function (RBF) neural network efficiently removes artifacts from the EMG signal, when compared to other types of neural networks. Table 2 demonstrates the comparison of performance parameters; *i.e.*, Mean Square Error (MSE) and correlation coefficient, among several neural network methods for EMG noise reduction [66,67]. In this case, a single, hidden layer of processing elements belongs to the RBF network which uses Gaussian transfer functions, rather than the standard sigmoidal functions employed by Multilayer Perceptron (MLP).

Table 2 represents the performance comparison of various neural networks. All the neural networks were trained to reduce the noises in the EMG signal using the training data. In addition, cross validation data were used to compare the efficiency of the learning ANN models in terms of solving the problem at hand.

Among these models, it compared: Multi-Layer Perceptron NN (MLP), Generalized Feed Forward NN (Gen FF), Modular NN (Mod NN), Jordan/Elman NN, and Recurrent Neural Network. The RBF network possesses several distinctive features, which makes it unique from other networks.

The general Equation (10), of this network is given below [68]:

(10) $$Y_{j}=\sum_{i=1}^{N}W_{\text{ij}}\phi \left(\left\|x-c_{i}\right\|\right)+\beta_{j}$$

Here, *ϕ*(‖*x*−*c_i_*‖) is the radial basis function of the hidden layer; ***W**_ij_* is the weight between ith hidden layer and jth output; ci = center vector; Yj and βj are the output of the network and bias value of the output jth neuron; and N = Number of nodes in the hidden layer.

Determining the number of neurons in the hidden layer is very crucial because the data learning capability in the RBF neural networks depends on its sufficiency [69]. Kale and Dudul demonstrated that a Focused Time-Lagged Recurrent Neural Network (FTLRNN)-based filter with a single, hidden layer elegantly removes noise from the EMG signal and gives reasonable accuracy [70]. According to their experimental study, Table 3 shows that compared to RBF NN and MLP, the FTLRNN model needs more time for training. However, the results of the Mean Square Error (MSE) and co-relation coefficient (r), and the visual inspection of modeling characteristics prove the FTLRNN model to be superior to the other two NNs. Here, the number of epochs is constant (1,000) for all three NNs. From the table, it can be observed that the FTLRNN model provides very low MSE and the high correlation coefficient. Therefore, FTLRNN is the best neural network to remove noise from an EMG signal.

An Artificial Neural Network is not a very common method for sEMG signal processing for noise reduction. However, in recent years several researchers have applied the different approaches of the ANN method to sEMG noise removal. By analyzing all of the approaches, the author recommends to use Jordan/Elman NN as a sEMG noise reduction approach. The advantage of the Jordan/Elman NN is that it is simple, speedy and is capable of generalization.

### 3.5. Independent Component Analysis (ICA)

The ICA algorithm has rapidly become one of the most prominent signal processing techniques. The ICA is a statistical method, which can assume the original signal from the mixture signal. P. Comon first proposed this method [71] and it is used for transforming an experimental multivariate random vector into components that are statistically independent from each other. In ICA there is no order of magnitude associated with each component, and the extracted components are invariant to the sign of the sources. Using this vector-matrix notation, the above mixing model is written as:

(11) x=As

**Figure 6.**
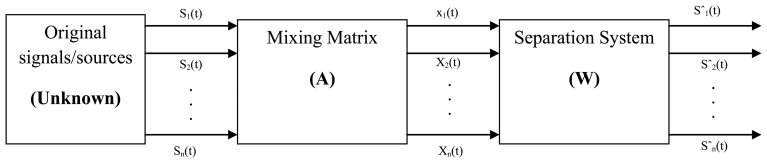
Blind source separation (BSS) block diagram [72].

Equation (11) represents an ICA model. Where *X* = [*x*_1_, *x*_2_…*x*_m_]*^T^* is an m vector of linear mixtures, *S* = [*s*_1_, *s*_2_,…,*s*_n_]*^T^* is an n-dimensional random vector of independent source signals, and A is full-rank m × n scalar linearly mixing matrix (n × m). Without knowing the source signals and the mixing matrix, a signal copy of the statistically independent sources s will be estimated from observed mixtures x. Figure 6 shows that the block diagram of the blind source separation technique.

In this figure s (t) are the sources. X (t) are the recordings sˆ (t) are the estimated sources, A is the mixing matrix, and W is the un-mixing matrix. Without non-Gaussianity, the estimation of the ICA model is not possible. ICA yields improvements above Principal Component Analysis (PCA), when signals do not display a Gaussian distribution [72]. It is suitable to separate the EMG signals from different sources when the assumptions below are fulfilled:

(i) Sources are independent at each time instant
(ii) Mixing matrix is linear and propagation delays of it are negligible
(iii) The sources are stationary and do not change with time
(iv) The signals are non-Gaussian
(v) The electromyographic (EMG) artifacts are statistically and mutually independent.

Consequently, ICA is a feasible method for source separation and decomposition of an EMG signal. Nowadays it is widely used to separate and remove noise sources from EMG and to decompose EMG signals into a maximum number of independent components. There are different types of ICA algorithms; some of them are used for processing the EMG signal, such as the Fast ICA algorithm, the Joint Approximate Diagonalization of Eigen-matrices (JADE), and the Infomax Estimation or maximum likelihood algorithm. The Fast ICA algorithm is a very popular method due to its simplicity, fast convergence and satisfactory results.

Hyvarinen introduced new contrast (or objective) functions for ICA based on the minimization of mutual information first [73]. There are two types of fast ICA algorithms: Fixed-point algorithm for one unit, and Fixed-point algorithm for several units. The Fast ICA algorithm could be performed at the beginning of each iteration, in order to solve overlaps and cancellations between MUAPs. It solves the low signal-to-noise ratio, which is the main complication in surface EMG signal decomposition [74]. Nakamura *et al.* reported that ICA is a very useful technique for decomposing sEMG signals into Motor-Unit Action Potentials (MUAPs) originating from different muscle sources. Fast ICA could provide much better discrimination of the properties of Motor-Unit Action Potential Trains (MUAPTs) for sEMG signal decomposition (*i.e.*, waveforms, discharge intervals, *etc.*) than PCA [75]. Fast ICA is a type of algorithm that successfully isolates power-line components from EMG signals. However, the performance of Fast ICA fluctuates quickly and few components obtained by ICA decomposition are inverted—a major problem when automatically decomposing EMG signals. Cardoso firstly proposed the JADE algorithm [76], which is more effective than Fast ICA for decomposing sEMG signals [73]. The JADE algorithm is based on the principle of computing several cumulant tensors, which are a generalization of matrices [77]. Firstly, Zhou *et al.* examined the feasibility of ICA based on an Information maximization (Infomax) algorithm for obtaining more information of the active motor units. Infomax ICA was unable to isolate all the MUAP trains due to time delays and the variances in shape between the surface action potentials detected at the different electrode locations. Furthermore, blind source separation techniques addressing a more complex convoluted mixing model are required for obtaining accurate firing rate information [78]. Bell and Sejnowski first introduced the Maximum Likelihood (ML) algorithm by using the stochastic gradient method [79]. The estimation of this algorithm is based on the fact that no prior information is available. Furthermore, Garcia *et al.* demonstrated that the JADE ICA could be used successfully for solving overlaps of MUAPs. In each iteration of the algorithm, the action potentials of one motor unit (MU) could generally be separated from the others. They showed that the JADE algorithm is more efficient than Fast ICA. JADE's performance is not strongly affected by added noise. However, inter-channel delay is the main drawback of this method [80].

In this section, the authors have reviewed some of the more prevalent approaches to ICA along with their potential benefits when applying them to EMG signals. The author has concluded that the ICA-based filtering procedure provides successful results in removing ECG artifacts and power-line noise (PLI), due to its largely independent signal-to-noise ratio, and because of its subtle effects on frequency content.

## 4. EMG Features

Because of the various noises and artifacts detected among EMG signals, required information remains an amalgam inside the raw EMG signals. However, if these raw signals are used as an input in sEMG classification, the efficiency of the classifier decreases. To improve the performance of the classifier, researchers have been using different types of EMG features as an input to the classifier. To achieve optimal classification performance, the properties of EMG feature space (e.g., Maximum Class separability, robustness, and the computational complexity) should be taken into consideration [81]. There are three types of EMG features in different domains: time domain, frequency domain and time-frequency domain features. Hudgins *et al.* developed time domain features of the sEMG. They used mean absolute value (MAV), mean absolute value slope, slope sign changes (SSC), waveform lengths (WL) and zero crossings (ZC) for representing myoelectric patterns [82–84]. These features are termed as ‘the Hudgins feature’. A carefully selected set of input features provides a higher classification rate than the raw signal [85,86]. In the quest to improve, the accuracy of myoelectric signal pattern classification Englehart *et al.* compared time domain (TD) features used by Hudgins [82] with the time frequency domain features (TFD) [87]. Based on the results of the classification error, they showed that feature based on Wavelet packet transform (WPT) was the most effective method. Time-frequency domain features are effective feature sets especially for transient myoelectric signal pattern classification. Due to the high dimensionality and high-resolution problem of time-frequency representation, dimensionality reduction is often a necessary complement to feature extraction [88]. Features based on Mean Frequency (MNF), Median Frequency (MDF), Mean Peak Frequency (PKF), Mean Power (MNP), Time-to-peak Force (TTP), Spectral Moments, Frequency Ratio (FR), Power Spectrum Ratio (PSR), and Variance of Central Frequency (VCF) are not good in EMG signal classification [89]. Table 4 shows the commonly used sEMG feature extraction method.

## 5. Classification

**Figure 7.**
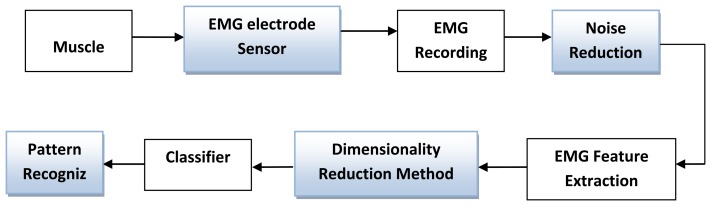
Block diagram of the process of EMG classification system.

An efficient means of classifying electromyography (EMG) signal patterns has been the interest of many researchers in the modern era. There are different types of classifiers, which are effectively used for different EMG applications, such as Artificial Neural Network (ANN), fuzzy classifier, Linear Discriminant Analysis (LDA), Self-Organizing Map (SOM) and Support Vector Machines (SVM) [89]. The raw EMG signal is represented as a feature vector in the feature extraction process, which is used as an input to the classifier. Because raw EMG signals directly feed to the classifier, they are not practical due to the randomness of the EMG signal. To avoid overloading the classifier, features were reduced in the dimension, using different dimension reduction methods. Dimensional reduction methods decrease the burden of the classifier and computational time. PCA is a more well-known method than other methods of dimension reduction [90]. Wavelet-based feature set reduced in dimension by principal components analysis greatly improves the classification accuracy in myoelectric-controlled prosthesis applicaion [91]. However, Chu *et al.* proposed a linear-nonlinear feature projection method by combining PCA with Self-Organizing Feature Map (SOFM). This method simplifies the structure of the classifier and provides greater classification performance compared to using only PCA [92]. In the dimension reduction method, the PCA and the Linear Discriminant Analysis (LDA) are well known. However, this method takes more computational time for solving the eigenvalue problem. The estimation method of LDA is a Simple Fisher Linear Discriminant Analysis (Simple-FLDA) that also can be used for the dimension reduction method. This algorithm takes less time to calculate the eigenvector because it does not use a matrix [93]. The amalgamation of TD features set with FLDA technique provides a good balance between robustness of the algorithm and computational efficiency. Accurate classification of EMG signal has great advantage on prosthetic control, which improves the quality of life of persons with limb deficiency. SVM and LDA classifiers are currently very popular amongst the researcher for the prosthetic control application due to their simple implementation and ease of training [94,95]. Figure 7 represents the main components of the EMG pattern recognition or classification method.

The success of the electromyogram classification system highly depends on the quality of the selected and extracted features [96]. Feature extraction step in the classification system increase information density of the signal [94]. Moreover, assessing and developing efficient dimensionality reduction and classifier methods are suggested for perfect EMG pattern recognition.

Many researchers have highlighted the neural network classifier in EMG pattern recognition because it can represent both linear and nonlinear relationships taken from data being modeled. ANNs are non-linear statistical data modeling tools that are inspired by the structure of biological neural networks and that are able to process an EMG signal. Del and Park suggested that ANN is a suitable technique for real-time applications of EMG [97]. ANN can precisely recognize the myoelectric (MES) signal. Data obtained by this unsupervised learning technique are then automatically targeted and presented to a Multilayer Perceptron-type Neural Network (MLP NN) [98]. The output of the neural network approach represents a quantity of preferred enervated muscle stimulation over a synergy [13]. In 1993, Tsuji *et al.* proposed an error back propagation-type neural network for the classification of six-forearm motions by using entropy [99]. Motion classification from the EMG signals is useful in fields such as control of multifunctional powered prosthesis, human-assisting robots or rehabilitation devices, and virtual reality.

**Figure 8.**
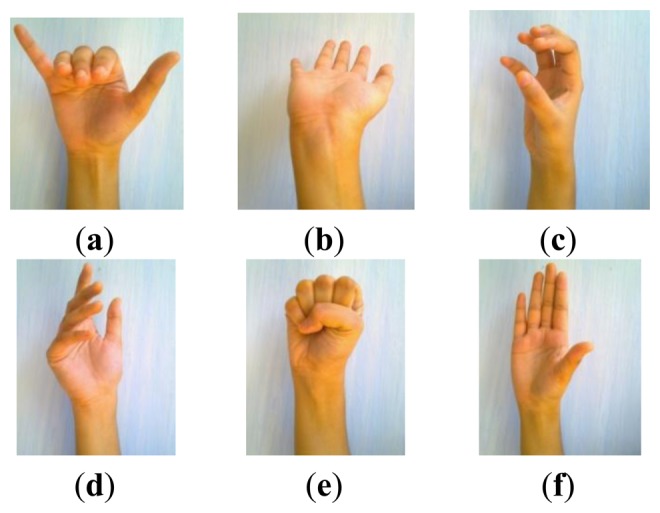
Six motions in the order of (**a**) flexion; (**b**) extension; (**c**) supination; (**d**) pronation; (**e**) hand grasping and (**f**) hand opening [100].

A new EMG pattern discrimination method, called the Recurrent Log-Linearized Gaussian Mixture Network (R-LLGMN), and based on the Hidden Markov Model (HMM), was proposed by Bu *et al.* [100]. For prosthetic control, they used this method and showed successful forearm motion (Figure 8) discrimination capability and accuracy, which was better than LLGMN and back propagation ANN (BPNN). As depicted in Table 5, they showed that the most excellent discrimination rate and approximately 0 standard deviation is obtained by using the R-LLGMN method among all three methods.

**Figure 9.**
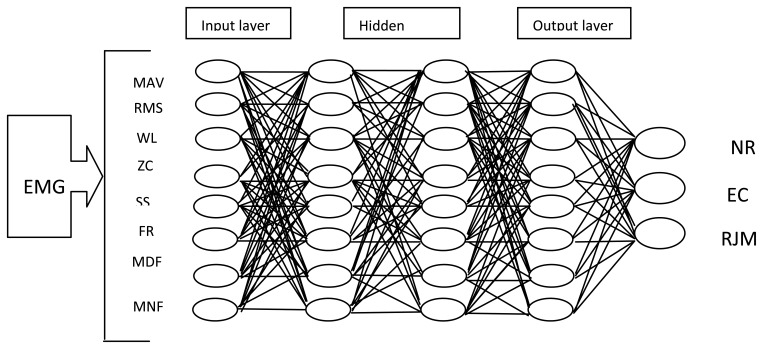
Schematic diagram of back propagation artificial neural networks (BPANN) with Levenberg-Marquardt algorithm [101].

Moreover, Wei *et al.* classified three EMG steady patterns—the normal (NR) pattern, the eye closing (EC) pattern, and the rhythmic jaw movement (RJM) pattern—by using BPANN with the Levenberg-Marquardt algorithm [101]. They used this classifier to generate five control commands for a simulated Intelligent Wheelchair. Figure 9 shows the block diagram of this algorithm.

On the other hand, ICA is a feasible method for source separation and decomposition of surface electromyogram (sEMG). Naik *et al.* examined four algorithms, Fast ICA, JADE-ICA, Infomax-ICA and Temporal Décor-relation Source Separation (TDSEP) ICA, for identifying subtle wrist actions. Table 6 represents the comparison between the various types of ICA algorithms [102].

TDSEP is an ICA algorithm based on the simultaneous diagonalization of several time-delayed correlation matrices. From the table it is observed that it provided the best performance and gave an overall efficiency of 97%. Use of ICA alone is not suitable for sEMG due to the nature of sEMG distribution and order ambiguity. Naik *et al.* proposed a novel method (Multi Run ICA) which is a combination of the mixing matrix and network weights to classify the sEMG recordings. This approach is able to overcome the ambiguity problems [103].

**Figure 10.**
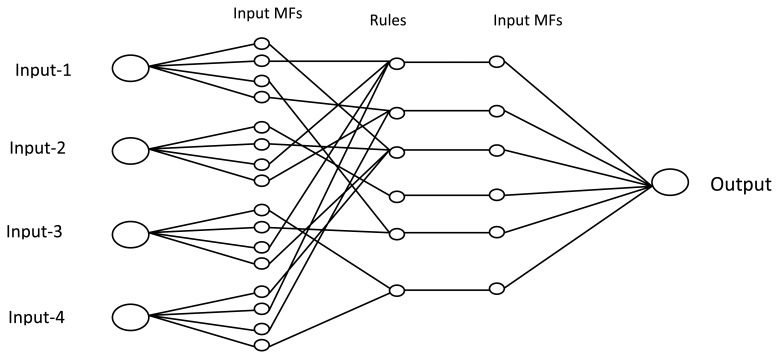
Structure of the fuzzy system with four inputs and one output [104].

Fuzzy logic systems have more advantages for bio-signal classification. Due to such biological signal characteristics as non-repeatable and stochastic, fuzzy logic is an advantageous technique in biomedical signal classification. Fuzzy logic methods are superior to neural network-based approaches because of their simplicity and insensitivity to over-training. The insufficient number of patterns interferes with the current sEMG, which repeatedly deepens by the inaccuracy of the instrumentation and analytical system. In order to resolve these difficulties, Khezri *et al.* suggested an Adaptive Neuro-Fuzzy Inference System (ANFIS) to detect hand gestures [104]. The fuzzy system initially fuzzifies the inputs to values at interval [0, 1], using a set of membership functions (MF's) [105–107]. Then, by using the IF-THEN rule it is derived by using fuzzy logic. The fuzzy rules can be represented Equation (12) as Ri: If x1 is MFi1 and/or x2 is MFi2 and/or … xj is MFj, then zi is:

(12) $$Z_{i}=\text{si}0+\text{si}1x1+\ldots .+\text{sijxj}$$

where Ri (i = 1, 2,…, l) denotes the ith fuzzy rule, xj (j = 1, 2,…, n) is the jth input, Zi is the output of the ith fuzzy rule, sij coefficients are the constants that are determined after training the fuzzy system, and, finally, MFij is the jth fuzzy MF of an antecedent for the ith rule. Figure 10 shows the Structure of the fuzzy system with four inputs and one output.

Based on the level of complexity and the change in hand movement and rate of precision, the ANFIS proves to be better than ANN. Table 7 shows the percentage of specificity and sensitivity measured in both ANFIS- and ANN-based methods [104].

The classification of electromyography (EMG) signals is also very important for detecting diseases. In clinical diagnosis, the simplicity, speed and reliability of classification are essential. The EMG signals from disabled patients or patients with different neurological diseases such as Parkinson's, Huntington's, Amyotrophic Lateral Sclerosis, *etc.* are very different from healthy EMG signals. All these types of patients have neurologic movement disorders and they could have different muscle structures. Therefore, many researchers have been working on EMG signals from these types of patients’ muscle tissue, recognizing it for monitoring the progression of the various diseases. Several research studies are also being carried out on neuromuscular fatigue (muscles that are not able to generate force or power) EMG. Furthermore, an accurate and computationally efficient means of classifying fatigue electromyographic signal patterns has been the subject of considerable research in recent years, which is most applicable in sports science.

**Figure 11.**
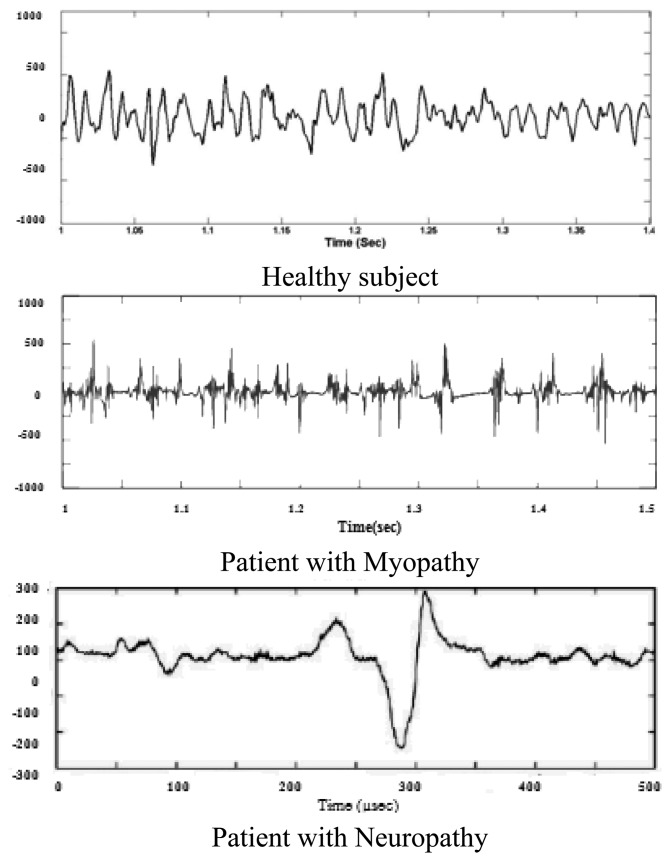
Three types of EMG signals; here y-axis represents amplitude (μV) [108].

Subasi *et al.* developed two classifier Feed-forward Error Back-propagation Artificial Neural Networks (FEBANN) and Wavelet Neural Networks (WNN) for diagnosing EMG patterns [108]. WNN is a neural network where a discrete wavelet function is used as a node activation function in a hidden layer. They used an autoregressive spectrum of EMG as an input to the input layer of FBANN with three discrete outputs representing normal, myopathic or neurogenic disorder. These three EMG patterns are shown in Figure 11.

In 2012, Christodouloua *et al.* used three classifiers, namely the statistical K-Nearest Neighbor (KNN), the Self-Organizing Map (SOM) and the Support Vector Machine (SVM) for classifying neuromuscular disorders (20 normal, 11 myopathy and nine neuropathy subjects) [109]. They first used multi-scale amplitude modulation-frequency modulation (AM-FM) features as an input to these classifiers. In their study, Gaussian Radial Basis Function was used in the SVM classifier. They proved that SVM provided the best diagnostic performance among all three classifiers. However, the learning speed of SVM was slow [110].

Subasi and Kiymik used the time-frequency methods such as STFT, Wigner-Ville Distribution (WVD) and Continuous Wavelet Transform (CWT), which have been used as pre-processing techniques. ICA was also used to reduce the dimension of feature vectors. Then, the extracted features of the EMG signal were used as an input to the Multilayer Perceptron Neural Network (MLPNN), which could be used to detect muscle fatigue. They showed that ANN with ICA separates EMG signals from healthy and fatigued muscles. By avoiding the spectral estimation, the problems of the conventional Fourier spectral variables deriving method is overcome by this method. Time-frequency methods do not assume quasi-stationarity or linearity in the order for this method to be appropriate for non-stationary signals. Muscle fatigue is automatically detected by this method [111].

Moreover, for classifying EMG signals, Sezgin used higher order spectra [112]. A bispectrum analysis (which belongs to the higher order spectra class) extracts the phase information from an EMG signal, which is called Quadratic Phase Coupling (QPC). These QPCs were fed into the Extreme Learning Machine (ELM) algorithm in order to separate abnormal activities from normal activities. The main advantage of ELM over the traditional learning methods is that it is capable of training and testing data fast and with a high accuracy. Therefore, this method may also be useful and applicable for a disease monitoring system. Table 8 represents the performance comparison among machine learning classification methods, such as ELM, Support Vector Machine (SVM), Logistic Regression (LR), Linear Discriminant Analysis (LDA) and Artificial Neural Network (ANN). A summary of electromyography pattern recognition techniques in different applications is presented in Table 9.

## 6. Discussion

This study showed that several undesired signal sources (extrinsic factors, inherent noise in electronic equipment, motion artifacts, ambient noise) can be attenuated to a great extent by using an active electrode. However, this basic technique is not sufficient for the abovementioned noise elimination problem. Researchers have used different types of processing techniques for canceling these noises. Proper use of these techniques can increase EMG signal quality to where the signal becomes much more accurate, simple, reliable and steady. Based on the studies reviewed, the wavelet transform and higher order spectra employed in the processing (noise reduction and significant information extract) method are optimal.

The study also described the use of the electromyography pattern recognition method, which is very important in different applications, such as rehabilitation devices, prosthetic arm/leg control, assistive technology, symptom detection for neuromuscular disorder, and so on.

In case of a disease monitoring system, two major criteria are applicable—one is robustness and reliability, and another is accuracy of diagnosis. Based on these criteria, the SVM classifier (where multi-scale Amplitude Modulation and Frequency Modulation (AM-FM) histogram features are used as an input) is suggested for classifying electromyography signals. AM-FM features can capture instantaneous variations in amplitude, frequency and phase of the electromyography signal. For real-time control of a robotic arm or leg, surface electromyographic (EMG) signal classification is also an important issue. On the other hand, if the number of EMG channels and features increases, the number of control commands of the classifier also increases. A large number of features (especially time domain and time scale feature vector) which extract the significant but different types of information from electromyography also provide improved classification results. Dimensionality reduction methods, Principle Component Analysis (PCA), and Linear Discriminant Analysis (LDA) methods are recommended if a huge number of features are used as input to the classifier. The main advantage of using the method is that the computational complexity of classifiers is allayed greatly. Dimensionality reduction methods should transform the data to a space vector with low dimensions and keep maximum information of the signal.

Furthermore, for increasing the classification accuracy, a combination of processing methods and pattern recognition techniques is strongly recommended. This combination method may be helpful to increase the classification accuracy without having to use too many muscle positions. The findings of this study are tabularized in Table 10, below.

## 7. Conclusions

A raw EMG signal contains more important information regarding the nervous system in useless form. The aim of this paper was to give detailed information about clearing up commonly associated noises and artifacts from EMG signals, and to explore the various methodologies for analyzing the signals. This study emphasized the algorithms and methodologies used for detecting, processing and classifying EMG signals, and discussed their advantages and disadvantages. This comparison of methodologies will help researchers encounter the perfect method for analyzing EMG signals, which is required in medical and physiological applications, such as diagnosis of neurological problems, biomedical and biochemical research, prosthetic arm control and end-user applications. It is the hope of this study to derive a clear and concise view of EMG processing methods for removing noise and to initiate improvements on current pattern recognition techniques.

## Figures and Tables

**Table 1.** List of 324 wavelet functions from 15 wavelet families.

**Wavelet Family**	**Wavelet Subtypes**	**No**
Haar	db1	1
Daubechies	db2-db45	2–45
Coiflet	coif1-coif5	46–50
Morlet	morl	51
Complex Morlet	cmor	52–147
Discrete Meyer	dmey	148
Meyer	meyr	149
Mexican Hat	mexh	150
Shannon	shan	151–200
Frequency B-spline	fbsp	201–260
Gaussian	gaus	261–267
Complex Gaussian	cgaus	268–275
Biorthogonal	bior	276–290
Reverse Biorthogonal	rbio	291–305
Symlet	sym	306–324

**Table 2.** Comparison of different types of Neural network [66].

**Sr no**	**Type of ANN**	**Hidden Layer Configuration**	**Correlation Coefficient(r)**	**Minimum MSE Criterion**		

				**Training**	**Crossvalidation**	**Testing**
01	MLP	05,05,07	0.627751035	0.0100858	0.02468346	0.00336508
02	Gen FF	05,05,07	**0.636240018**	**0.0095010**	0.01897900	0.00467948
03	Mod NN	05,05,07	0.636114324	0.0115398	0.02638402	0.00299289
04	Jor/elman NN	05,05,07	0.627025792	0.00994905	0.025520535	0.003213602
05	Recurrent NN	05,05,07	0.616395357	0.00997408	0.024154242	0.003366557
06	RBF network	05,05,07	**0.634935685**	**0.00996188**	0.025991453	0.003341636

**Table 3.** Comparison of all the NN architectures on test dataset [70].

**NN Model**	**Transfer Function in Output Layer**	**Learning Rule**	**Mean Square Error (MSE)**	**Correlation Coefficient (r)**	**Epochs**	**Time Elapsed (μs)**	**%Error**
MLP	Tanh	Momentum	0.02501 (noise)	0.78114 (noise)	1,000	19.16	253
			0.02482 (EMG)	0.58433 (EMG)			

FTLRNN	Linear	Momentum	0.000067 (noise)	0.99950 (noise)	1,000	14	10
			0.000048 (EMG)	0.99939 (EMG)			

RBF	Linear	Levenberg Marquardt (LM)	0.02470 (noise)	0.78414 (noise)	1,000	8.3	293
			0.02482 (EMG)	0.58509 (EMG)			

**Table 4.** Mathematical representation of widely used sEMG feature extraction methods.

**Feature Extraction**	**Mathematical Equation**
Integrated EMG(IEMG)	$\text{IEMG}=\sum_{n=1}^{N}\left|x_{n}\right|$
	Here *N* denotes the length of the signal and *x_n_* represents the sEMG signal in a segment.

Mean Absolute Value (MAV)	$\text{MAV}=\frac{1}{N}\sum_{n=1}^{N}\left|x_{n}\right|$

Modified Mean Absolute Value 1 (MMAV1)	$\text{MMAV}1=\frac{1}{N}\sum_{n=1}^{N}w_{n}\left|x_{n}\right|$
	$w_{n}=\{\begin{array}{cc} 1, & \text{if}\;0.25N\leq n\leq 0.75N\\ 0.5, & \text{otherwise} \end{array}$

Modified Mean Absolute Value 2 (MMAV2)	$\text{MMAV}2=\frac{1}{N}\sum_{n=1}^{N}w_{n}\left|x_{n}\right|$
	$w_{n}=\{\begin{array}{c} 1,\;\text{if}\;0.25N\leq n\leq 75N\\ \frac{4n}{n},\;\text{if}\;0.25N\leq n\\ \frac{4\left(n-N\right)}{n},\;\text{if}\;0.75N\leq n \end{array}$

Simple Square Integral(SSI)	SSI=∑n=1N|xn|^2

Variance of EMG (VAR)	VAR=1N−1∑n=1Nxn^2

Root Mean Square (RMS)	RMS=1N∑n=1Nxn^2

Waveform Length (WL)	$WL=\sum_{n=1}^{N}\left|x_{n+1}-x_{n}\right|$

Willison Amplitude (WAMP)	$\text{WAMP}=\sum_{n=1}^{N}f\left|x_{n+1}-x_{n}\right|$
	$f\left(x\right)=\{\begin{array}{c} 1,\;\text{if}\;\text{x}\geq \text{threshold}\\ 0,\;\text{otherwise} \end{array}$

Log detector (LOG)	$\text{LOG}=e^{\frac{1}{N}\sum_{n=1}^{N}\text{log}\left|x_{n}\right|}$

Slope Sign Change (SSC)	$\text{SSC}=\sum_{n=2}^{N}f\left[\left(x_{n}-x_{n-1}\right)\times (x_{n}-x_{n+1})\right]$
	$f\left(x\right)=\{\begin{array}{c} 1,\;\text{if}\;\text{x}\geq \text{threshold}\\ 0,\;\text{otherwise} \end{array}$

Zero crossing (ZC)	$ZC=\sum_{n=1}^{N-1}[\text{sgn}(x_{n}\times x_{n+1})\cap^{}\left|x_{n}-x_{n+1}\right|\geq \text{threshold}]$
	$\text{sgn}=\{\begin{array}{c} 1,\;\text{if}\;\text{x}\geq \text{threshold}\\ 0,\;\text{otherwise} \end{array}$

Multi-scale amplitude modulation–frequency modulation (AM–FM)	$f\left(k\right)=\sum_{n=1}^{M}a_{n}\left(k\right)\text{cos}\emptyset_{n}(k)$
	Here n = 1, 2,…M indexes the AM–FM components, a_n_ represents the nth instantaneous amplitude, and ϕ_n_ represents the nth instantaneous phase. Here, AM–FM components are extracted over a dyadic filter bank.

**Table 5.** Discrimination results of five subjects (A, B, C, D and E) [100].

**Subject**	**A**	**B**	**C**	**D**	**E**
R-LLGMN Mean ± SD (%)	99.06 ± 0.00	89.32 ± 0.37	93.04 ± 0.11	93.49 ± 0.00	92.75 ± 0.00
LLGMN (Mean ± SD (%))	94.00 ± 5.50	82.83 ± 0.00	88.50 ± 0.04	88.67 ± 0.15	89.26 ± 0.14
BPNN (Mean ± SD (%))	73.41 ± 7.86	46.52 ± 12.3	44.20 ± 10.4	69.79 ± 9.97	69.17 ± 7.00

**Table 6.** Performance of four types ICA algorithm (percentage) for isometric hand gesture Identification [102].

**Number of Participants**	**Middle and Index Finger Flexion**	**Little and Ring Finger Flexion**	**All Finger Flexion**	**Finger and Wrist Flexion Together**
Raw EMG	60%	60%	60%	60%
Infomax	80%	80%	80%	80%
JADE	85%	85%	85%	85%
Fast ICA	90%	90%	90%	90%
TDESP	97%	97%	97%	97%

**Table 7.** Performance comparison between ANFIS- and ANN-based methods [104].

**Classifier**	**Specificity**	**Sensitivity**
ANFIS	92%	94.67%
ANN	86.6%	92.2%

**Table 8.** Performances of the ANN, SVM, LR, LDA and ELM learning machines [112].

**Model**	**Training Process Time (s)**	**Testing Process Time (s)**	**Accuracy (%)**
ANN	32.25	1.18	98.20
SVM	1.80	0.20	96.15
LR	0.10	0.05	97.50
LDA	0.09	0.04	97.25
ELM	0.07	0.005	99.75

**Table 9.** Summary of different EMG classification system.

**Feature**	**Classifier**	**Classfication Type**	**Electrode Sensor Placement**	**Correctness**	**Reference**
FFT	NN (Feature dimensionality reduction by (Simple-FLDA)	Recognize Wrist motion	FCR & FCU (Four electrode)	94%	Oyama and Mitsukura [93]

MAV, SSCs, and AR model coefficients of the signal, ZC	Adaptive Neuro-fuzzy interference system (ANFIS)	Six classes of hand movement	Extensor digitorum, ECR, PL and FCU	92%	Khezri & Jahed [104]

MAV,RMS, VAR, SD, ZC, SSC & WL	BPANN with Levenberg-Marquardt training algorithm	Hand motion pattern	Hand	89.2%	Ahsan *et al.* [85]

WPT	MLP(Feature dimensionality reduction by SOFM + PCA)	Multifunction myoelectric hand control	*Extensor digitorum*, Extensor carpi radialis, PL and FCU (Four channel)	97%	Chu, J.U *et al.* [92]

FFT	Fuzzy interference system (FIS)	Hand motion recognition for controlling Robot hand	Hand	90%	Uchida *et al.* [106]

RMS	SVM	Eight classes of hand movement for realtime control of a robotic arm.	Flexor carpi radialis, FCU, *Pronator teres*, *Bracioradialis*, ECD, *Anconius*, *Pronator quadrates*	92–98%	Shenoy *et al.* [107]

RMS,Entropy	BPANN (Gradient-descent algorithm)	Four hand gestures recognition for human-computer interaction	Forearm, Abductor *Pollicies longus* (four channel)	97.5%	Rajesh *et al.* [86]

ARM and EMG histogram	CKLM	Control of a multi-degrees-of-freedom prosthetic hand.	PL, EDC,FCU, FDS,FDP	93.54%	Yi-Hung [105]

Entropy	Error backpropagation type neural networks	Six Motion discrimination	Forearm (four paired electrode)	90%	Tsuji *et al.* [99]

Force information *F*EMG, Entropy	R-LLGMN	Six motion discrimination	Forearm (six channel)	-	Nan Bu *et al.* [100]

RMS	BPANN	Classify six different hand gestures	Flexor carpi radialis, FCU, FDS, *Bracioradialis* (Four electrode channels)	99%	Naik *et al.* [103]

AM-FM	KNN	Classified neuromuscular disorder	*Biceps brachii* muscle	58%	Christodouloua *et al.* [109]
	
	SOM			60%	
	
	SVM			78%	

AR	WNN	Classified neuromuscular disorder	*Biceps brachii* muscle	90.7%	Subasi *et al.* [108]
	
	FEBANN			88%	

Vector elements extracted by STFT	MLPNN with Levenberg-Marquardt (L-M) and gradient descent (GDA) algorithms (Feature dimensionality reduction by ICA).	Muscle fatigue detection	Right *biceps brachii* muscle	88.5%	Subasi A. *et al.* [111]
	
SPWVD				90%	
	
CWT				91%	


Quadratic phase coupling (QPC)	Extreme Learning Machine Algorithm (ELM)	Classify the EMG signals (an aggressive action or a normal action)	Right biceps & triceps, Left biceps & triceps, right & left thigh, right & left hamstring (8 channel)	99.75%	Sezgin, N. [112]

AR	SVM	Diagnosis of neuromuscular diseases	Biceps and Hypothenar eminence	-	Güler *et al.* [110]

IEMG-Integrated EMG, WPT-Wavelet packet Transform, FFT-Fast-Fourier Transform, STFT-Short time Fourier Transform, SPWVD-Smoothed Pseudo-Wigner-Ville Distribution, CWT- Continuous wavelet transform, AR-Autoregressive analysis, MAV-Mean amplitude value, RMS-Root mean square, VAR-Variance value, SD-Standard deviation, ZC-Zero crossing, SSC-Slope Sign Changes, WL-Wave length, REC-Recurrent Rate, Pacc-Power, Wmax-Wavelet coefficient, Samp En-Entropy, ARM-Autoregressive model, FMN-Frequency mean, FMD-Frequency median, FR-Frequency ratio, PL-*Palmaris longus*, EDC-*Extensor digitorum communis*, FCU-*Flexor carpi ulnaris*, FDS-*Flexor digitorum superficialis*, FDP-*Flexor digitorum profundus*, FEBANN-Feed forward error backpropagation artificial neural networks, WNN-wavelet neural networks, BPNN-Back propagation neural network

**Table 10.** Summary of most important methods.

**Methods**	**Characteristics**
Wigner-Ville Distribution (WVD)	WVD exhibits excellent localization properties. It is very noisy, which is the major limitation of this method.

Wavelet Transform (WT)	WT has the capability of multiresolution problem. It is able to deal with multicomponent signals because it is not affected by cross-term. The stationary signal is assumed, it is the main restriction of WT.

Artificial Neural Network (ANN)	ANN can represent both linear and nonlinear relationships. Exhibit mapping potentialities, it can learn to map a set of inputs to a set of outputs and precisely detect data. The complexity of the network structure increases if the number of input dimensions increases.

Higher order statistics (HOS)	HOS is very useful in the detection and characterization of non-linearities of mechanisms that generate time series via phase relations of their harmonic components. The HOS characterizes the non-Gaussianity in a signal very well because the HOS of Gaussian signals are statistically zero. It contains both amplitude and phase information. HOS are translation invariant.

Empirical Mode Decomposition (EMD)	EMD method is able to deal with non-stationary and non-linear data. It can decompose any complicated time series data precisely. The main difficulties of the EMD method is to implement the best spline. EMD algorithm is very sensitive for noise. Enhanced empirical mode decomposition is noise-assisted version and it is more robust.

Independent Component Analysis (ICA)	Sources e.g independent component must be non-Gaussian for ICA which is the fundamental restriction of this method. It is sensitive to high-order statistics in the data, not just the covariance matrix. It delivers a more probable set of data, which helps to locate the data concentration in n-dimensional space.

Fuzzy Logic	It is very simple and is insensitive to over training. The most important characteristics of the fuzzy logic system is that it can tolerate a certain degree of contradiction in the data.
